# The neurotoxic effects of ampicillin-associated gut bacterial imbalances compared to those of orally administered propionic acid in the etiology of persistent autistic features in rat pups: effects of various dietary regimens

**DOI:** 10.1186/s13099-015-0054-4

**Published:** 2015-03-22

**Authors:** Afaf El-Ansary, Ramesa Shafi Bhat, Sooad Al-Daihan, Abeer M Al Dbass

**Affiliations:** Biochemistry Department, Science College, King Saud University, P.O.Box 22452, , Zip code 11495 Riyadh, Saudi Arabia; Medicinal Chemistry Department, National Research Centre, Dokki, Cairo Egypt

**Keywords:** Autism, Ampicillin, Propionic acid, Neurotoxicity, Gut microbiota, Dietary regimens

## Abstract

**Hypothesis:**

A healthy gut with normal intestinal microflora is completely disrupted by oral antibiotics. The byproducts of harmful gut bacteria can interfere with brain development and may contribute to autism. Strategies to improve the gut microflora profile through dietary modification may help to alleviate gut disorders in autistic patients.

**Method:**

Sixty young male western albino rats were divided into six equal groups. The first group served as the control; the second group was given an oral neurotoxic dose of propionic (PPA) (250 mg/kg body weight/day) for three days. The third group received an orogastric dose of ampicillin (50 mg/kg for three weeks) with a standard diet. Groups 4, 5 and 6 were given an orogastric dose of ampicillin and fed high-carbohydrate, high-protein and high-lipid diets, respectively, for 10 weeks. Biochemical parameters related to oxidative stress were investigated in brain homogenates from each group.

**Result:**

The microbiology results revealed descriptive changes in the fecal microbiota of rats treated with ampicillin either alone or with the three dietary regimens. The results of PPA acid and ampicillin treatment showed significant increases in lipid peroxidation and catalase with decreases in glutathione and potassium compared with levels in the control group. A protein-rich diet was effective at restoring the glutathione level, while the carbohydrate-rich diet recovered lipid peroxidation and catalase activity. In addition, the three dietary regimens significantly increase the potassium level in the brain tissue of the test animals. Lactate dehydrogenase was remarkably elevated in all groups relative to the control. No outstanding effects were observed in glutathione S-transferase and creatine kinase.

**Conclusion:**

The changes observed in the measured parameters reflect the neurotoxic effects of PPA and ampicillin. Lipid peroxide and catalase activity and the levels of glutathione and potassium are satisfactory biomarkers of PPA and ampicillin neurotoxicity. Based on the effects of the three dietary regimens, a balanced diet can protect against PPA or ampicillin-induced neurotoxicity that might induce autistic traits. These outcomes will help efforts directed at controlling the prevalence of autism, a disorder that has recently been associated with PPA neurotoxicity.

## Background

Human beings and their gut microbiota are in a symbiotic relationship, and a “super organism” that includes both the human organism and microbes has been recently proposed by SommerandBäckhed [1]. It is now clear that bidirectional signaling between the gut and the brain, mainly through the vagus nerve, the so-called “microbiota-gut-vagus-brain axis”, is vital for maintaining homeostasis and may be also involved in the etiology of certain mental disorders [2]. Shaping of the microbiota occurs in parallel with brain development, and the microbiota-gut-brain axis is therefore considered to be a key player during neurodevelopment. Accordingly, early life events during the initial colonization and microbiota development can determine general and mental health in later life [3,4]. Disruptions during these critical periods of dynamic microbiota-host interaction have a great potential to alter brain-gut signaling, affect health throughout life, and increase the risk of neurodevelopmental disorders.

It appears that alterations in the gut microbiota during childhood and adolescence could be susceptible to environmental factors, such as the use of antibiotics, stress, poor diet, and infection, which could result in dysbiosis and have a potentially negative impact on general and mental health, leading to the development of brain disorders later in life. Although antibiotics are common environmental insults with a profound impact on intestinal microbiota, data on antibiotic use as a risk factor for a subsequent neurodevelopmental abnormality are scarce [5-7]. Although the pathophysiology of Autism Spectrum Disorder remains unknown, a number of metabolic pathways appear to be modulated in this disorder and have been highlighted as possible candidate disease mechanisms, which under a given genetic susceptibility, may alter neurological development in early childhood [8,9]. Gastrointestinal symptoms have long indicated that gut bacteria might play a role in the pathophysiology of ASD,and indeed, various studies have shown that the gut microbiota is altered in ASD,although there is little agreement in the literature as to which bacteria might be involved [10,11].

The gut microbiota usually shows a remarkable capacity to recover once a course of antibiotics is stopped. Sandler et al. [12] showed that 8 weeks of treatment with vancomycin, which actively inhibits Clostridium bacteria, improved communication and behavior scores in autistic children, but these scores deteriorated again upon discontinuation of the antibiotic antibiotic which ascertain the role of Clostridium in the etiology of autism.

In addition, gut microbiota provide an important mediator of the bioavailability and toxicity of environmental pollutants (e.g.,heavy metals and propionic acid). These microorganisms and their metabolites have a strong impact on pH, oxidative balance, detoxification enzymes, and xenobiotic-metabolizing and transporting host proteins, all of which may strongly influence the bioavailability of toxins in the gut lumen [13].

Intestinal bacteria are recognized as potentially important factors that contribute to the increased prevalence of diseases associated with antibiotic use, such as hypersensitivity pneumonitis (HP) and autism [14,15]. Finegold [16] proposed that certain antibiotics may play key roles in modifying the gut bacterial flora adversely, allowing the overgrowth of harmful bacteria that are normally suppressed by an intact normal gut flora or probiotics. Antibiotics (such as penicillin, cephalosporin and clindamycin)can also disturb normal flora to allow the overgrowth of *Clostridium difficile,* which in turn has been associated with the development of autism [17]. These bacteria are spore-formers capable of resisting antibacterial drugs. If the antibiotics are discontinued, the spores germinate and produce toxins and metabolites, including short-chain propionic acid (PPA), which has recently been reported to induce persistent biochemical and behavioral autistic features in rat pups [18-20].

Autistic children with a low level of glutathione usually have amplified levels of toxic metals, such as mercury (Hg) and lead (Pb) [21]. To a certain extent, a low glutathione level decreases the ability of the body to excrete Hg and lead (Pb) [22,23]. The augmented use of certain oral antibiotics, such as ampicillin, also decreases the ability to excrete toxic metals into the feces [24]. Ampicillin has been reported to reduce the excretion of inorganic mercury to 26% and that of total mercury to 60% in the feces compared with the excretion levels of control rats, which can be attributed to altered gut flora. Altered gut flora due to the administration of oral antibiotics has also resulted in the complete inability to excrete mercury in rats [25].

These findings motivate our interest to test and compare the neurotoxic effects of ampicillin to those induced by propionic acid as a metabolite of propionibacteria. Propionic acid producers were induced post-ampicillin treatment of rat pups. The study was also extended to evaluate the effects of high-protein (HP), high-carbohydrate (HC), and high-lipid (HL) diets on the neurotoxic effects of ampicillin. The three dietary regimens used in the present study were not isocaloric but were used to investigate the differences related to their nutritional importance in the presence of ampicillin-induced neurotoxicity regardless of their varied caloric loads.

Because metabolic activity is tightly coupled with neuronal activity, the measured parameters were selected carefully to reflect changes in brain metabolism [26]. These parameters include energy requirements-related parameters (lactate dehydrogenase, and creatine kinase) [26], pro-oxidant/antioxidant status parameters (MDA, GSH, GST, and catalase) [27], and voltage-gated channels-related parameter (K^+^) [28].

## Results

Tables 1 and 2 demonstrate the induced changes in fecal microbiota either in ampicillin-treated or ampicillin treated rats fed various dietary regimens. Six weeks after the antibiotic treatments, the diversity of gastrointestinal flora remained altered, while certain bacterial species disappeared (e.g., *Enterobacter cloacae*) and certain propionibacteria were introduced (e.g., *Klebsiella pneumonia* and *Proteus vulgaris*)*.* In addition, the fungal growth of *Candida albicans* was observed on ampicillin treatment. *Candida tropicalis, Rhizobium radiobacter,* and *Enterococcus* species were observed in rats fed an HLD compared to the other groups except the animals fed an HPD, which demonstrates the induced growth of *Rhizobium radiobacter.* After 6 weeks of ampicillin treatment (Table 2), it was observed that the fungal growth of both *C. albicans* and *Streptococcus* species had ceased, while the rest of the microbial profile was more or less the same.

**Table 1:** **Descriptive changes in the fecal flora immediately after an orogastric dose of ampicillin (50 mg/kg for three weeks)**

**Bacteria**	**Control**	**Ampicillin**	**A + CHO**	**A + oil**	**A + protein**
*E. coli*	+	+	+	+	+
*E. cloacae*	+	-	-	-	-
*Staphylococcus spp.*	+	+	+	+	+
*B. species*	-	-	+	+	-
*A.fumigatus*	-	-	-	-	-
*P. mirabilis*	-	-	-	-	-
*K. pneumonia*	-	+	+	+	+
*P. vulgaris*	-	+	+	+	+
*C.albicans*	-	+	-	+	-
*Streptococcus*	-	-	+	+	-
*C.tropicalis*	-	-	-	+	-

**Table 2:** **Descriptive changes in the fecal flora six weeks after orogastric ampicillin (50 mg/kg for three weeks)**

**Bacteria**	**Control**	**Ampicillin**	**A + CHO**	**A + oil**	**A + protein**
*E. coli*	+	+	+	+	+
*E.cloacae*	+	-	-	-	-
*Staphylococcus spp.*	+	+	+	+	+
*B. species*	-	+	-	+	+
*A.fumigatus*	-	-	-	-	-
*P.mirabilis*	-	-	-	-	-
*K. pneumonia*	-	+	-	+	+
*P.vulgaris*	-	+	+	+	+
*C.albicans*	-	-	-	-	-
*Streptococcus*	-	-	-	-	-
*C.tropicalis*	-	-	-	+	-
*R.radiobacter*	-	-	-	+	+
*Enterococcus species*	-	-	-	+	-

Table 3 demonstrates the significant depletion of glutathione with PPA acid and ampicillin treatments compared to control. While PPA produced a 20.35% decrease in glutathione (P˂0.033), ampicillin was more effective and produced a 33.82% reduction in GSH levels (P˂0.001). However, PPA induced more lipid peroxidation in the rat pup brains than did ampicillin, with recorded values of 206.48% and 122.94%, respectively. Among the two dietary regimens, the protein-rich diet was effective in restoring GSH levels in the brains of ampicillin-treated rat pups. However, the HPD induced more lipid peroxides (MDA), while the HCD showed ameliorating effect with lipid peroxide levels that were not significantly different (P˂0.204) from the untreated control animals.

**Table 3:** **Mean ± S.D. of all the measured parameters in PPA-treated, ampicillin-treated and animals fed various diets compared to control**

**Parameter**	**Group**	**N**	**Mean ± S.D.**	**%Change**	**P value** ^**a**^	**P value** ^**b**^	**P value** ^**c**^
Glutathione (μg/ml)	Control	10	67.02 ± 14.29	100.00			0.001
	Pro-acid	10	53.38 ± 11.98	79.65	0.033		
	Ampicillin (A)	10	44.35 ± 9.97	66.18	0.001	0.084	
	A + CHO	10	43.89 ± 6.51	65.49	0.001	0.041	
	A + Protein	10	72.46 ± 14.94	108.12	0.416	0.006	
	A + Oil	9	52.33 ± 16.34	78.08	0.052	0.874	
Lipid peroxides (MD μmoles/ml)	Control	10	0.057 ± 0.005	100.00			0.001
	Pro-acid	10	0.118 ± 0.037	206.48	0.001		
	Ampicillin	10	0.070 ± 0.010	122.94	0.002	0.003	
	A + CHO	10	0.067 ± 0.022	116.46	0.204	0.002	
	A + Protein	10	0.119 ± 0.017	208.41	0.001	0.933	
	A + Oil	9	0.157 ± 0.031	274.43	0.001	0.025	
Glutathione S-transferase activity (U/ml)	Control	10	227.06 ± 50.39	100.00			0.011
	Pro-acid	10	190.62 ± 28.97	83.95	0.063		
	Ampicillin	10	256.14 ± 21.94	112.81	0.120	0.001	
	A + CHO	10	235.17 ± 50.96	103.57	0.725	0.027	
	A + Protein	10	245.27 ± 45.71	108.02	0.408	0.005	
	A + Oil	9	240.74 ± 26.27	106.02	0.476	0.001	
Catalase activity (U/dL)	Control	10	6.59 ± 1.89	100.00			0.001
	Pro-acid	10	9.31 ± 3.48	141.31	0.047		
	Ampicillin	10	7.53 ± 2.09	114.28	0.306	0.186	
	A + CHO	10	4.80 ± 3.03	72.84	0.130	0.006	
	A + Protein	10	10.56 ± 3.77	160.24	0.011	0.452	
	A + Oil	9	8.91 ± 2.41	135.15	0.032	0.774	
Potassium (mmol/L)	Control	10	10.10 ± 1.97	100.00			0.001
	Pro-acid	10	8.48 ± 1.81	83.95	0.072		
	Ampicillin	10	9.18 ± 2.26	90.84	0.342	0.456	
	A + CHO	10	15.19 ± 3.34	150.34	0.001	0.001	
	A + Protein	10	13.74 ± 1.89	136.00	0.001	0.001	
	A + Oil	9	15.31 ± 3.55	151.61	0.001	0.001	
Creatine kinase (U/L)	Control	8	3900.01 ± 495.26	100.00			0.001
	Pro-acid	10	3948.36 ± 422.41	101.24	0.826		
	Ampicillin	9	4138.42 ± 353.99	106.11	0.267	0.306	
	A + CH	9	3824.58 ± 618.43	98.07	0.787	0.614	
	A + Protein	7	3945.15 ± 159.99	101.16	0.813	0.983	
	A + Oil	7	3062.79 ± 257.84	78.53	0.002	0.001	
Lactate dehydrogenase (μmoles)	Control	10	1842.16 ± 420.30	100.00			0.001
	Pro-acid	10	2525.06 ± 322.42	137.07	0.001		
	Ampicillin	10	2547.80 ± 232.25	138.30	0.001	0.858	
	A + CH	10	2773.58 ± 208.62	150.56	0.001	0.056	
	A + Protein	8	2512.36 ± 505.38	136.38	0.007	0.949	
	A + Oil	6	2564.08 ± 381.26	139.19	0.004	0.830	

^**a**^P value between control group and other groups.

^**b**^P value between Pro-acid group and other groups.

^**C**^P value between all groups.

N.B. Variations in the numbers of investigated samples in each group are due to the insufficiency of certain samples or the death of animals during the experimental period (e.g., animals fed a high-lipid diet).

Although PPA induced a 16% reduction in the activity of glutathione S-transferase, this effect was not significantly different from that of the control. Ampicillin treatment together with the dietary regimens showed more or less similar effects compared to those of the control. Catalase, another antioxidant enzyme, was induced with both treatments and showed a significant difference with PPA treatment (P˂0.047) but not ampicillin treatment (P˂0.306) compared to control. The catalase activity in rat pups fed an HCD after ampicillin treatment was similar to that of the control (P˂0.306) but significantly different from that of PPA-treated animals (P˂0.006).

Lactate dehydrogenase was remarkably elevated in all groups compared to that of control. Ampicillin induced more or less similar changes compared to those in PPA-treated animals (P˂0.858).

Creatine kinase was the least affected parameter. It exhibited levels similar to those of the control in PPA- and ampicillin-treated animals, as well as those fed an HCD or HPD.

Potassium was significantly lower in PPA-intoxicated rats, while ampicillin did not induce a significant alteration in the K^+^ level. The three dietary regimens significantly increased the K^+^ level (P˂0.001).

**Figure 1:**
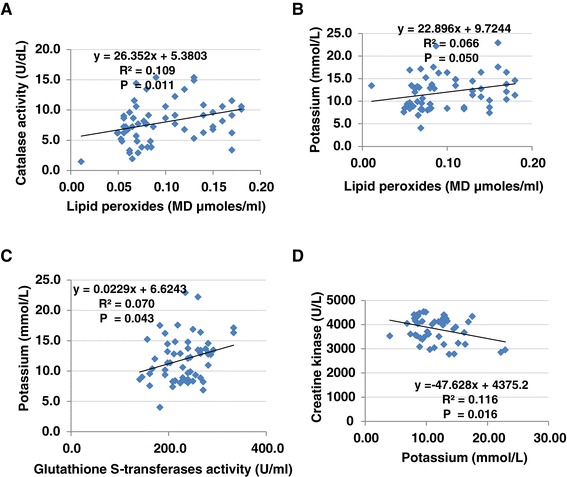
**Pearson’s correlations between eight measured parameters. A**. catalase activity verses lipid peroxides, **B**. potassium verses lipid peroxides, **C**. potassium verses glutathione-s-transferases, **D**. creatine kinase verses potassium.

Table 4 and Figure 1 present Pearson’s correlations between the measured parameters. It is clear that lipid peroxide (MDA) was positively correlated with catalase and potassium and negatively correlated with CK. In addition, potassium was positively correlated with glutathione S-transferase and negatively correlated with CK.

**Table 4:** **Pearson’s correlations between the measured parameters**

**Parameters**	**R (pearson correlation)**	**Sig.**	
Lipid peroxides vs. Catalase activity	0.329^*^	0.011	P^a^
Lipid peroxides vs. Potassium	0.256^*^	0.050	P^a^
Potassium vs. Glutathione S-transferase activity	0.264^*^	0.043	P^a^
Potassium vs. Creatine kinase	−0.340^*^	0.016	N^b^

Correlation is significant at the 0.01 level.

^*^Correlation is significant at the 0.05 level.

^**a**^Positive correlation.

^**b**^Negative correlation.

Table 5 shows the ROC analysis with the area under the curve (AUC), specificity and sensitivity of the measured parameters under various experimental conditions. Because AUC is a measure of the overall performance of a diagnostic test, the overall diagnostic performance of the various treatments of the present study can be compared by comparing their AUCs, specificity and sensitivity.

**Table 5:** **ROC curve of all parameters in all groups**

	**Group**	**Area under the curve**	**Best cutoff value**	**Sensitivity%**	**Specificity%**
Glutathione (ug/ml)	Pro-acid	0.504	56.755	60.0%	60.2%
	Ampicillin	0.731	54.345	90.0%	55.1%
	A + CHO	0.784	45.800	80.0%	77.6%
	A + Protein	0.834	63.880	80.0%	83.7%
	A + Oil	0.583	42.130	44.4%	82.0%
Lipid peroxides (MD μmoles/ml)	Pro-acid	0.695	0.068	100.0%	36.7%
	Ampicillin	0.691	0.083	100.0%	57.1%
	A + CHO	0.695	0.089	100.0%	51.0%
	A + Protein	0.734	0.089	100.0%	69.4%
	A + Oil	0.931	0.135	88.9%	88.0%
Glutathione S-transferase activity (U/ml)	Pro-acid	0.846	216.145	90.0%	75.5%
	Ampicillin	0.716	221.350	100.0%	44.9%
	A + CHO	0.502	247.350	50.0%	65.3%
	A + Protein	0.578	278.645	30.0%	89.8%
	A + Oil	0.560	216.145	88.9%	40.0%
Catalase activity (U/dL)	Pro-acid	0.612	9.360	50.0%	75.5%
	Ampicillin	0.528	9.360	90.0%	32.7%
	A + CHO	0.813	5.000	70.0%	89.8%
	A + Protein	0.740	10.680	50.0%	89.8%
	A + Oil	0.660	7.440	88.9%	54.0%
Potassium (mmol/L)	Pro-acid	0.820	10.485	100.0%	69.4%
	Ampicillin	0.806	8.890	80.0%	83.7%
	A + CHO	0.804	12.790	90.0%	67.3%
	A + Protein	0.711	10.780	100.0%	53.1%
	A + Oil	0.802	11.145	100.0%	54.0%
Creatine kinase (U/L)	Pro-acid	0.580	3315.665	100.0%	25.0%
	Ampicillin	0.732	4155.140	66.7%	78.0%
	A + CHO	0.518	4065.610	66.7%	58.5%
	A + Protein	0.515	4122.180	100.0%	44.2%
	A + Oil	0.947	3522.195	100.0%	83.7%
Lactate dehydrogenase (μmoles)	Pro-acid	0.551	2033.600	100.0%	22.7%
	Ampicillin	0.539	2295.660	90.0%	36.4%
	A + CH	0.797	2435.740	100.0%	52.3%
	A + Protein	0.543	2589.005	75.0%	50.0%
	A + Oil	0.628	2803.150	50.0%	85.4%

Table 6 presents multiple regression analysis using lipid peroxide as the dependent variable. It is clear that among the measured parameters, CK, catalase and lactate dehydrogenase show significance as independent variables with a P˂0.001 and an R^2^ value of 0.251.

**Table 6:** **Multiple regression using the Stepwise method with lipid peroxides (MD** μ**moles/ml) as a dependent variable**

**Predictor variable**	**Beta**	**P value**	**Adjusted R** ^**2**^	**Model**	
				**F value**	**P value**
reatine kinase (U/L)	−0.339	0.01	0.251	6.350	0.001
Catalase activity (U/dL)	0.314	0.017			
Lactate dehydrogenase (μmoles)	0.304	0.02			

## Discussion

The gut microbiota of mammals play a key role in modulating host physiology. These effects are mostly associated with immunity and nutrient intake. Antibiotic usage strongly affects gut microbial composition and metabolism, thereby impacting human health. Understanding the mechanisms underlying this important interaction remains a major research goal [29].

Tables 1 and 2 demonstrate alterations in the gut microbiota of ampicillin-treated rat pups. Although the microbiology technique applied in this study did not measure the growth of *Clostridia* species, it was able to effectively detect the overgrowth of *Klebsiella pneumonia,* which is similar to *Clostridia* species in that both are propionibacteria and produce propionic acid as a metabolite [30,31].The growth of *K. pneumonia* in ampicillin-treated rat pups could be supported by the fact that *K. pneumonia* has β-lactamase enzyme and is thus resistant to ampicillin as a β-lactam antibiotic [32]. Interestingly, the reported overgrowth of *Proteus vulgaris* in ampicillin-treated rats could be easily related to PPA production. In a study conducted many years ago by Sherman and Shaw [33], *Proteus vulgaris* was reported to be an opportunistic human pathogen that accelerated the activity of PPA bacteria, which include *Clostridia* species and *K. pneumonia*. This phenomenon could explain the role of PPA in inducing biochemical autistic features in ampicillin-treated rat pups.

GSH depletion increases the cellular vulnerability toward oxidative stress, particularly in children due to their lower GSH levels [34,35]. The risk due to impairment of the detoxification capacity in infants is higher because certain toxic environmental factors that induce oxidative stress accumulate in the placenta and are found at higher concentrations in developing infants than in their mothers.

Table 3 demonstrates glutathione depletion as a feature of PPA and ampicillin neurotoxicity, with ampicillin being more toxic than PPA and showing a more significant reduction compared to that of the control. Associated with the glutathione levels, which are reduced in PPA- and ampicillin-stressed animals, Table 3 also shows that in these animals, glutathione levels can be restored by dietary supplementation with a protein-rich diet but not a carbohydrate- or lipid-rich diet. This finding could indicate that rats with GSH deficiency may present a model for realistic toxicological testing, one that is more closely related to the clinical condition of a critically ill patient on drug orantibiotics treatment. The recorded effect of a protein-rich diet in restoring the brain GSH level can be supported by the previous work of Micke et al [36], in which they reported that short-term (14 days) oral supplementation (45 g/day) with whey protein formulas increased the plasma GSH levels in glutathione-deficient patients with advanced HIV infection. However, the inefficiency of HCD and HLD for restoring GSH in ampicillin-treated rats is in good agreement with a recent study of Alzoubi et al. [37] reporting that a high-lipid/high-carbohydrate diet (HLCD) induces oxidative stress that presents as high-lipid peroxides and concomitant low GSH levels and results in neuronal damage and interference with synaptic transmission leading to a decline in cognitive function.

Table 3 also demonstrates lipid peroxides (MDA) elevation as a metabolic marker of PPA and ampicillin neurotoxicity, with ampicillin being less toxic than PPA and showing a reduced but still significant elevation compared to the control. This finding is supported by the recent work of El-Ansary et al. [38], in which GSH depletion and MDA elevation were observed as biochemical autistic features in a rodent model of autism. While an HCD demonstrated a similar change in lipid peroxidation compared to that of the control, the HFD, and unexpectedly, the HPD induced more lipid peroxidation in ampicillin-treated animals. This effect of an HPD could find support in the previous work of CalderónGuzmán et al. [39], which proved that MDA was decreased in several brain areas of malnourished rats fed a low-protein diet (7%). The effect of an HLD on MDA reported in the present study is consistent with the recent work of Amin et al. [40], in which the consumption of an HLD for12 weeks was proven to be adequate to increase the level of MDA, a marker of oxidative stress, in the brains of rats. Induced lipid peroxidation in HLD-fed ampicillin-treated rats can be explained according to a gut-brain-liver neuronal mechanism and lipid-sensing system. Lipids in the lumen of rats fed a lipid-rich diet stimulate the release of cholecystokinin (CCK) from intestinal cells lining the mucosa of the duodenum [41]; the CCK then binds to the CCK-A receptor in the gastrointestinal system and indirectly regulates hepatic glucose synthesis. Lipid-induced CCK can activate the release of glutamate (the principal excitatory neurotransmitter) from vagal afferent terminals [42]. This neuronal axis represents one of the first lines of metabolic defense against nutrient excess, providing metabolic balance by lowering glucose production upon nutrient exposure. This suggested involvement of CCK in the attenuation of the lipid-sensing mechanism and the reported elevation of MDA in HLD-fed ampicillin-treated rats can be supported by the current work of Tamás et al. [43], in which MDA was significantly elevated in the pancreatic tissue of a CCK-treated group compared to untreated animals. Interestingly, the suggested involvement of a gut-brain neuronal mechanism in ampicillin-treated rats fed an HLD can easily be related to glutamate excitotoxicity, which is a mechanism that has recently been highlighted in the etiology of autism. Wyeth et al. [44] reported that CCK induced an up-regulation of excitatory glutamatergic neurons and a down-regulation of inhibitory gamma amino butyric acid (GABA) ergic neurons in mice with spontaneous seizures. This finding raises the possibility that, in the present study, the suggested amplification of CCK in response to a lipid-rich diet could explain the epileptic seizures observed in most autistic patients and indicate the possibility of avoiding them through dietary changes. The observed effects of HCD and HPD reported in the present study are inconsistent with previous reports from Kitabchi et al. [45] that a low-carbohydrate (rather than a high-protein) diet is more efficacious in lowering MDA in obese, premenopausal non-diabetic women.

Antioxidant enzymes are an appropriate indirect measure to evaluate the pro-oxidant/antioxidant status associated with PPA- and ampicillin-induced toxicity. The activity of GST decreased in PPA-treated rats, while it was increased in ampicillin-treated rats and in ampicillin-treated rats fed HC, HP and HL diets. The remarkable increase in GST in the brains of antibiotic-treated rats and antibiotic-treated rats fed a modified diet could be related to the induction of GST in the medium culture as a type of defense against ampicillin toxicity. This explanation could be supported by the work of Shaffer et al. 46], which reported the efficiency of GST in decreasing the activity of many antibiotics, including ampicillin, resulting in increased values of the minimum inhibitory concentrations (MIC). A single exposure caused the leakage of proteins from brain mitochondria, a high rate of lipid peroxidation, and a significant increase in cytosolic antioxidant enzymes, thus demonstrating that antioxidant defenses act as an early manifestation of antibiotic neurotoxicity that is remarkably affected by diet. This explanation is consistent with the reported ampicillin-induced neurotoxicity noted in very low birth weight neonates, which has been attributed to immature transport mechanisms and renal immaturity, as well as increased permeability of the blood brain barrier [46]. The decrease of GST activity in PPA-treated rats is a reproducible neurotoxic effect of this short-chain fatty acid [38].

Catalase is an important enzyme in the elimination of H_2_O_2_from tissues, and it has been suggested that catalase and not glutathione peroxidase (GPX) is the major antioxidant enzyme responsible for H_2_O_2_ degradation [47]. Because H_2_O_2_ induces catalase expression, it is likely that PPA-treated rats, ampicillin-treated rats and ampicillin-treated rats fed either an HPD or HLD diet have elevated levels of this ROS. This result could be related to the pathological effects of PPA and ampicillin because similarly increased levels of catalase were found in H_2_O_2_-stressed Chinese hamster fibroblasts, A549 human lung adenocarcinoma cells and U87MG glioblastoma cells [48,49]. The increased catalase activity observed in PPA-treated rats does not contradict the observed decrease in catalase activity that has previously been reported as an autistic feature in PPA-treated rat pups [38].This finding is expected because it is well known that for in vivo systems, if the oxidative stress is not very strong or very prolonged, the catalase activity increases, but if it is persistent or its level is very high, the protein damage becomes profound, and a decrease in the catalase activity may occur (either via direct oxidative damage of the catalase molecules, via oxidative stress-altered gene expression, or both). This assumption could be supported by the depletion of GSH and elevation of MDA (two important markers of oxidative stress) reported in the present study and in our previous study. Rats fed a HC diet plus ampicillin show significantly reduced catalase activity. This finding is supported by the work of Francini et al. [50], which reported significant reductions of 24% and 18% in the total GSH content and CAT activity, respectively, in the livers of rats fed a high-fructose diet.

The results of the present study show the LDH activity increased to 40-50% in the 5 groups relative to control (p < 0.001), whereas CK activity was significantly decreased only in the ampicillin-treated rats fed a high-lipid diet (p < 0.002). As LDH is an important cytoplasmic enzyme that regulates energy metabolism in the cell, the remarkable increase in the activity of this enzyme shows that PPA and ampicillin both induce changes in the plasma membrane permeability with consequent cellular damage. The neurotoxicity of both agents can be explained by an increase in LDH activity causing a low pH due to high lactate production. Lactic acidosis could in turn induce the denaturation of integral and structural proteins, injuring neurons [51] With the exception of HCD; there was no significant dietary effect on LDH activity. The remarkable increase in LDH activity in rats fed an HCD compared to the tested groups could be explained by the idea that LDH, as a glycolytic enzyme, is activated in response to the increase in glucose in an attempt to enhance its catabolism. This assumption is consistent with a previous study by Walton [52], in which the activation of certain glycolytic enzymes, including LDH, was recorded in response to a high-carbohydrate/low-protein diet in the rainbow trout *Salmogairdneri*.

Biochemically, HLD-induced disturbances in metabolism cause an increase in the flux of free fatty acids (FFA) into a variety of tissues. This response mediates brain disturbances through a reduction in the ability of brain to respond to various stressors and a loss of the capacity to repair damaged brain cells. In the present study, the decrease in CK activity in animals fed an HLD after ampicillin treatment is not in good agreement with the previous work of Amin et al. [40], in which an HLD induced an increase in CK secondary to the induction of glycolytic enzymes. The significant decrease in CK in rats fed an HLD could be attributed to the synergistic effect of the antibiotic. Although ampicillin alone did not induce a significant impairment in CK, rats fed an HLD post ampicillin treatment exhibited a highly significant decrease in CK compared to those of control untreated and unfed rats. The suggested synergistic effect of HLD can be supported by the previous work of Ribeiro et al. [53], which demonstrated that HLD aggravates the toxicity of hydrochlorothiazide (HCTZ) in treated rat brains.

Voltage-gated K^+^ channels (Kvs), together with a presynaptic Ca^2+^ influx, control the release of neurotransmitters [54], and these channels play a critical role in glucose homeostasis. Blocking Kvs reduces IL-2 and tumor necrosis factor production, resulting in improved autoimmune encephalitis and the inhibition of microglial-mediated neuronal death in experimental models [55,56]. The reported low level of K^+^ in PPA-neuro-intoxicated rats could indicate the importance of maintaining K^+^ within a controlled concentration range for normal brain synaptic function. The significant increase in K^+^ reported in ampicillin-treated rats could be attributed to the potent effect of ampicillin under various nutritional conditions on activation of the Na^+^/K^+^ ATPase and the total ATPase activity [40].

The positive correlations of lipid peroxides (MD, as a marker of oxidative stress) with catalase, GST and K^+^ and its negative correlation with CK(as a marker of energy metabolism) (Table 4) show that oxidative stress is an etiological mechanism involved in the neurotoxicity of PPA and ampicillin.

In addition to the AUC, the specificity and sensitivity values listed in Table 5 demonstrate the possibility of using GSH, MDA, GST and K^+^ as markers of PPA and ampicillin neurotoxicity. In addition, K^+^ was the most potent marker of ampicillin neurotoxicity,showing effectiveness when testing both toxic agents together in conjunction with the three dietary regimens.

## Conclusion

The present work proposes that ampicillin-induced neurotoxicity might be among the etiologies of late-onset autism due to alterations in gut microbiota and the induction of propionibacteria. Examining how disturbances in the gut flora can induce oxidative stress as a mechanism that remarkably affects the brain in rodent models of autism can reveal promising targets for the development of diagnostic markers of this disorder. As ampicillin is used to treat a wide variety of infections caused by bacteria, such as ear infections (which are commonly observed in autistic patients), bladder infections, and pneumonia, a good balanced diet rich in a variety of nutrients is crucial to ameliorate or avoid the neurotoxic effects of antibiotics, which are the most frequently used pharmaceuticals during brain development in cases of frequent infection.

## Methods

### Animals

The experimental assays for this study were performed on 60 young (approximately 21 days old) male western albino rats (45 to 60 g). Rats were obtained from the animal house of the Pharmacy College of King Saud University and were randomly assigned to six groups of ten rats each. The first group of rats received only phosphate buffered saline and were used as a control group (n = ten). The second group was given oral neurotoxic doses of PPA (250 mg/kg body weight/day for three days; n = ten) [57] and were referred to as the oral buffered PA-treated group. The third group received an orogastric dose of ampicillin (50 mg/kg for three weeks) with standard diet and referred to as the ampicillin group. Groups 4, 5 and 6 were given orogastric doses of ampicillin (50 mg/kg for three weeks). This dosage was appropriate and selected according to clinical guidelines and the diagnosis and treatment manual (30–100 mg/kg in humans) [58,59]. The ampicillin-treated animals were fed a high-carbohydrate, high-protein or high-lipid diet for 10 weeks. All groups of rats were individually housed under a controlled temperature (21 ± 1°C) with ad libitum access to food and water. All of the procedures described were reviewed and approved by the King Saud University animal ethical committee.

### Diet

The standard rats/mice chow consisted of 47% complex carbohydrates, 21% protein, 4.0% fat, 5.0% fiber, and 8.0% ash. The special diets characterized as high-carbohydrate and high-protein were made by mixing pulverized regular chow with sucrose and casein, respectively, and then pelleting. The pellets were baked at 55°C for 7 hours before use. The lipid-rich diet was made by adding corn oil to the regular chow.

### Tissue preparation

At the end of the feeding trials, the brain was dissected out and submitted to biochemical analysis. The brains isolated from sacrificed animals were washed, dissected into small pieces and homogenized in distilled water using a Teflon homogenizer. The homogenate was centrifuged at 3,000 g for 20 minutes to remove debris and kept at −80°C until further use.

### Biochemical analyses

The following biochemical estimations were made from the brain tissue samples. Glutathione was assayed by the method of Beutler et al. [60] using 5,5′-dithiobis 2-nitrobenzoic acid (DTNB) with sulfhydryl compounds to produce a relatively stable yellow color. Lipid oxidation was estimated by the formation of thiobarbituric acid reactive substances (TBARS) by the method of Ruiz-Larrea et al. [61]. Glutathione S-transferase activity (GST) activity was assessed using an assay kit (Biovision, USA) that was based upon the GST-catalyzed reaction between GSH, GST substrate, and CDNB (1-chloro-2,4-dinitrobenzene). The activity of catalase was determined by the method of Chance [62], in which the levels of catalase activity were expressed as μmoles of H_2_O_2_ dissociated/minute/dl. Lactate dehydrogenase was assayed using the lactate-to-pyruvate kinetic method described by Henry et al. [63]. An assay of creatine kinase was performed using the CK kit from the National Scientific Company (NSC) [64]. Potassium levels were measured by producing a turbid suspension in a protein-free alkaline medium by reaction with sodium tetraphenyl boron [65].

### Microbiological examination

The cecal contents of the all experimental groups were collected in sterile tubes and immediately stored at −20°C. The frozen tubes were then analyzed. The process of bacterial cultivation involves the use of optimal artificial media and incubation conditions to isolate and identify the bacterial etiologies of an infection as rapidly and accurately as possible. Fecal samples were cultured under aerobic and anaerobic conditions and continuously monitored for one week. Positive cultures were plated with appropriate media, and the species were identified by Scepter micro dilution and standard bacteriological techniques. All of the plates were examined after 24 and 48 h of incubation at 37°C. This work was performed in a microbiology lab at Almishari Hospital, Riyadh, KSA.

### Statistical analysis

The SPSS software program was used. The results were expressed as the mean ± SD, and all statistical comparisons were made using independent t-tests with P ≤ 0.05considered as significant. Receiver Operating Characteristic (ROC) analysis was performed as a comprehensive way to measure the effectiveness of the studied parameters in terms of either the neurotoxicity of PPA and ampicillin or the effects of the various dietary regimens. The area under the curve (AUC) provides a useful metric to compare various biomarkers. Whereas an AUC value close to 1 indicates an excellent predictive marker, a curve that lies close to the diagonal (AUC = 0.5) has no diagnostic utility. An AUC of 0.8-0.9 represents a good test, 0.7-0.8 indicates fair, 0.6-0.7 indicates poor, and 0.5-0.6 indicates a worthless test. AUC analysis is always accompanied by the relative values of specificity and sensitivity of the biomarker [66]. Multiple regression analysis using the Stepwise method was used to evaluate the relationship between MDA as a dependent variable and the remaining measured parameters as independent variables. The value of R^2^ usually indicates the percentage of the variance of the dependent variable associated with the regression of the recorded independent variables.
